# Plasmonic Resonance Coupling of Nanodisk Array/Thin Film on the Optical Fiber Tip for Integrated and Miniaturized Sensing Detection

**DOI:** 10.3390/s23084163

**Published:** 2023-04-21

**Authors:** Hao He, Xinran Wei, Yijin He, Yuzhang Liang, Yurui Fang, Wei Peng

**Affiliations:** School of Physics, Dalian University of Technology, Dalian 116024, China; hehaogttdjd@mail.dlut.edu.cn (H.H.); dutweixinran@163.com (X.W.); 19834040754@mail.dlut.edu.cn (Y.H.); wpeng@dlut.edu.cn (W.P.)

**Keywords:** plasmonic nanostructure, biosensing, structural transfer, fiber facet, surface plasmon resonance, mode coupling

## Abstract

Fiber-optic surface plasmon resonance (FOSPR) sensing technology has become an appealing candidate in biochemical sensing applications due to its distinguished capability of remote and point-of-care detection. However, FOSPR sensing devices with a flat plasmonic film on the optical fiber tip are seldom proposed with most reports concentrating on fiber sidewalls. In this paper, we propose and experimentally demonstrate the plasmonic coupled structure of a gold (Au) nanodisk array and a thin film integrated into the fiber facet, enabling the excitation of the plasmon mode on the planar gold film by strong coupling. This plasmonic fiber sensor is fabricated by the ultraviolet (UV) curing adhesive transferring technology from a planar substrate to a fiber facet. The experimental results demonstrate that the fabricated sensing probe has a bulk refractive index sensitivity of 137.28 nm/RIU and exhibits moderate surface sensitivity by measuring the spatial localization of its excited plasmon mode on Au film by layer-by-layer self-assembly technology. Furthermore, the fabricated plasmonic sensing probe enables the detection of bovine serum albumin (BSA) biomolecule with a detection limit of 19.35 μM. The demonstrated fiber probe here provides a potential strategy to integrate plasmonic nanostructure on the fiber facet with excellent sensing performance, which has a unique application prospect in the detection of remote, in situ, and in vivo invasion.

## 1. Introduction

Fiber-optic surface plasmon resonance (FOSPR) sensing technology has been proven to be one of the most feasible schemes for realizing biochemical sensing detection with high sensitivity, strong anti-interference ability, and label-free detection [1,2]. This sensing technology is becoming an excellent and indispensable platform in a wide variety of fields of life sciences, drug screening, environmental monitoring, and the like due to its prominent capability of remote and point-of-care detection [3,4,5,6]. Conventional FOSPR sensors, as miniaturized substitutes for the complex and bulk prism-coupled SPR sensing devices, achieve total reflection when configured with a metal film on the fiber core side without a cladding layer [7,8]. In these FOSPR sensors, SPR on the outer surface of the metal film is excited by the principle of total internal reflection in fiber, enabling the real-time and highly sensitive detection of the ambient surroundings by demodulation of the optical signals such as wavelength, the intensity around the resonance wavelength, and others [9]. Moreover, various functional materials such as nanoparticles [10,11], multilayer metal/dielectric layers [12,13], graphene [14,15], and polymers [16] are integrated on the surface of FOSPR sensors to improve their sensitivity and tunability, further expanding their practical applications. In addition, tilted fiber Bragg grating SPR sensors [17,18,19], as another common FOSPR sensor, can achieve SPR on the outer surface of metal film without removing cladding by the diffraction effect of tilted grating, exhibiting a wide range of applications in various detection fields such as disease biomarkers, heavy metal pollution, and the like.

Currently, FOSPR sensing devices capitalizing on flat plasmonic film are mainly achieved on fiber sidewalls. However, FOSPR sensors on thin film at the fiber end facet are rarely proposed [20,21]. Compared to fiber sidewall counterparts, fiber facet-integrated FOSPR sensors possess unparalleled superiority in the detection of in situ and in vivo invasion due to their extremely small sensing area. Additionally, their dip-and-read operation mode greatly reduces the time and complexity of operation [22,23,24]. In order to achieve the excitation of SPR on the fiber facet, some metallic nanostructures are patterned on the end face of the optical fiber through various micro-nano-processing technologies such as electron beam lithography (EBL), focused ion beam (FIB), or nanoimprint [25,26,27], which greatly promotes the development of FOSPR sensors. However, these preparation methods have some limitations such as processing difficulties and unavailability of batch manufacturing, which is not conducive to the production of FOSPR sensors in industry [28,29,30]. Consequently, there is an urgent need to develop a FOSPR sensor on flat plasmonic film at the fiber facet that can be easily mass-manufactured and still possess excellent sensing performance.

In this paper, we develop and experimentally demonstrate a plasmonic fiber probe consisting of a Au nanodisk array and thin film integrated into the fiber tip by UV curing adhesive transferring technology [31,32], which enables the excitation of plasmon mode on the uppermost Au film at the end facet of optical fiber. In the proposed plasmonic fiber probe, the bottom Au nanodisk array stimulates the enhanced Bloch-type surface plasmon polariton to tunnel to the top of the Au thin film to excite the plasmon mode supported by it [33]. The effect of the thickness of the top Au film on the bulk sensitivity of the proposed fiber probe is first investigated. As a result, a moderate bulk RI sensitivity of 137.28 nm/RIU is achieved with optimal structural parameters. Additionally, the surface sensitivity of this fiber probe is also investigated experimentally by layer-by-layer self-assembly PAH/PSS bilayers on top of the fiber end face, effectively consolidating the spatial localization of excited plasmon mode. Finally, various concentrations of bovine serum albumin (BSA) aqueous solution are detected by using the fabricated plasmonic fiber probe with a detection of limit as low as 19.35 μM, indicating its potential for practical biomolecular detection. In addition, compared with the traditionally indirect preparation process, the UV curing adhesive transfer technology, as an indirect approach, has the advantages of high-efficiency, high-quality, and low-cost fabrication of nanostructures on the optical fiber tip, and is expected to facilitate the mass production of fiber tip sensors.

## 2. Material and Method 

### 2.1. Material

The UV curing optical adhesive of NOA 81 was purchased from Thorlabs Inc. (Newton, MA, USA). Poly(allylamine) hydrochloride (PAH, Mw~65 kDa), poly(sodium-p-styrenesulfonate) (PSS, Mw~70 kDa), and sodium chloride (NaCl) were obtained from Sigma-Aldrich Inc. Bovine serum albumin (BSA, 66 kDa) and phosphate-buffered saline (PBS) solution (pH 7.4) were purchased from Sangon Biotech Inc. The multimode fiber used for sensing, purchased from Shenzhen Xinrui Optical Co., Ltd. (Shenzhen, China), has a core/cladding/buffer diameter of 400/440/650 µm with a numerical aperture (NA) of 0.22. The materials of fiber core, cladding, and buffer layer are high OH quartz, fluorine-doped quartz, and acrylic resin, respectively. The ultrathin AAO membranes used here are prepared by a two-step anodization process. Al foils are first cleaned and polished, then anodized in phosphoric acid for a sacrificial layer. The irregular anodic oxide layer is removed with a mixed acid solution before a second anodization. The thickness and pore size are adjusted by anodizing and corrosion time. A PMMA layer is added and Al substrates are removed with phosphoric acid. PMMA/AAO sheets are dissolved in acetone to leave the AAO membranes suspended. They are transferred to objective substrates and attached by quick-drying acetone [33]. Furthermore, BSA biomolecule solutions with a concentration ranging from 10^−8^ to 10^−4^ M are prepared with PBS buffer solution. A low concentration of BSA solution is obtained by diluting a high-concentration sample to improve the accuracy of solution preparation.

### 2.2. Plasmonic Nanostructure-Based Fiber-Optic Sensing Probe

**Figure 1:**
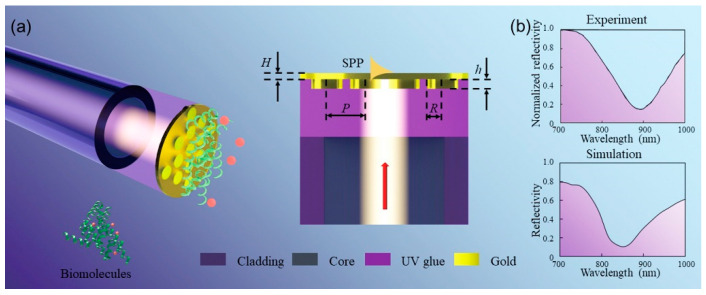
Schematic of the proposed nanodisk array/thin film-based fiber probe and its characteristic optical spectra. (**a**) Its three-dimensional drawing and cross-section clearly demonstrate the plasmonic coupled nanostructure of Au nanodisk array/thin film integrated on the fiber end face by UV curing adhesive, where the planar Au film resides on the top surface of the fiber probe. The corresponding structural parameters and the propagation direction of incident light (red arrow) are also indicated in (**a**). (**b**) Measured and simulated reflection spectra of nanodisk array/thin film-based fiber probe immersed in a pure water solution.

The proposed plasmonic nanostructure-based fiber probe is constructed by a triangular array of Au nanodisk on a nano-thick Au thin film reversely placed on the end face of fiber by UV curing adhesive. As a result, a flat Au surface is built atop the fiber probe, which is more convenient for molecular modification in sensing detection as compared to an uneven surface. As shown in Figure 1a, three-dimensional and cross-sectional schematics of the proposed plasmonic fiber probe are demonstrated, where the Au nanodisk array is arranged with a quasi-period (*P*) of about 450 nm, and the radius and height of the nanodisk and the thickness of top Au film are marked as *R*, *h*, and *H*, respectively. The incident light propagating along the fiber core (indicated by the red arrow) irradiates the surface of the Au nanodisk array embedded in UV curing adhesive, exciting the plasmon mode at the interface of the nanodisk array and UV glue by the diffractive coupling of the localized dipolar mode of nanodisk. The plasmon mode is then inspired at the top surface of the thin Au film through strong coupling. In the design of structural parameters and mechanism analysis of the involved plasmon mode, the finite-difference time-domain (FDTD) method is employed to calculate optical spectra and electromagnetic field distributions. In the simulation, the periodic boundary conditions are set along the *x* and *y* directions, and the perfectly matched layer boundary conditions are applied along the *z* direction. The finer mesh size of 2 nm × 3.46 nm × 1 nm is used for all numerical calculations. The permittivity of Au in the visible and near-infrared region is from Johnson and Christy [34], and the refractive index (RI) of UV curing adhesive is taken as the constant of 1.56. The experimentally measured reflection spectrum of the fabricated plasmonic nanodisk array/film-based fiber probe immersed in pure water solution is demonstrated in the top panel of Figure 1b, where there is one resonant dip at the 898 nm in the wide wavelength range from 700 nm to 1000 nm. Obviously, the measured reflection spectrum is similar to the simulated one (the bottom panel of Figure 1b) in accordance with the spectrum lineshape, as well as the position and number of resonant dips. There are also some deviations between them, which are attributed to the following three factors: first, the inconsistent structural parameters include inevitable surface roughness, different geometry dimensions, and the discrepancy of material parameters; second, the imperfectly periodic arrangement and randomly varied nanodisk radius with limited range is not considered in the simulation; third, the simulation only considers the case at normal incidence, whereas the experimental data are the result of spectral superposition within a limited angle range due to the existence of fiber, NA.

### 2.3. Experimental Fabrication and Characterization of the Nanodisk Array/Thin Film-Based Fiber Probe

**Figure 2:**
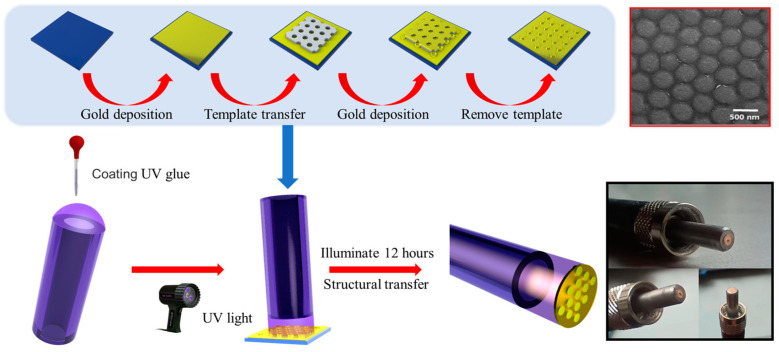
Schematic of the transferring preparation procedure for the designed plasmonic fiber probe by patterning nanodisk array/thin film structure. The entire fabrication procedure mainly consists of two processes: one is the designed nanostructure fabricated on the Si substrate by using an ultrathin porous AAO membrane (top panel); the other is the transferring of the designed nanostructure from the planar Si substrate to fiber end face using UV curing glue (bottom panel). The upper right inset shows an SEM image of the Au nanodisk array/thin film structure on the Si substrate. The bottom right inset shows photographs of the fabricated nanodisk array/thin film-based fiber probe.

The preparation procedure of the designed nanodisk array/thin film-based fiber probe is summarized in Figure 2, which mainly includes two processes: one is the fabrication of the designed nanostructure on a silicon (Si) substrate using an ultrathin porous AAO membrane (top panel); the other is the transferring of the designed nanostructure from the planar Si substrate to the fiber facet using UV curing glue (bottom panel). The specific preparation procedure is illustrated in detail as follows: first, a nano-thick Au thin film is deposited on a clean Si substrate by magnetron sputtering. A quasi-ordered porous alumina (AAO) ultrathin membrane with a lattice constant of ~450 nm and a thickness of ~300 nm is then transferred to the prepared substrate as a patterning mask. Subsequently, a 50 nm thick Au film is deposited on the Si substrate with an AAO mask. Next, the AAO mask is mechanically stripped with epoxy tape to naturally form a Au nanodisk array/thin-film nanostructure with the diameter of the nanodisk being in the range from 260 nm to 360 nm. The scanning electron microscopy (SEM) image in the top right inset of Figure 2 shows the experimentally fabricated nanodisk array/thin film structure on the Si substrate, where the presence of crystalline Au is further verified by the results of energy dispersive spectrometer elemental analysis (not shown here). Afterward, the UV curing epoxy of NOA 81 as an adhesion layer is attached to the polished fiber end face, then vertically touches the prefabricated nanostructure. The UV-curing epoxy is irradiated by UV light for 12 h until it is completely cured. Finally, the fiber tip is withdrawn from the transfer template. Due to the weak binding force between the Au thin film and Si wafer, the Au nanodisk array/thin-film nanostructure is detached from the Si substrate and remains on the fiber facet. The photographs in the bottom right inset of Figure 2 demonstrate the experimentally fabricated nanodisk array/thin film-based fiber probe. 

## 3. Results and Discussion

### 3.1. Theoretical Explanation and the Superiority of the Designed Nanostructure

**Figure 3:**
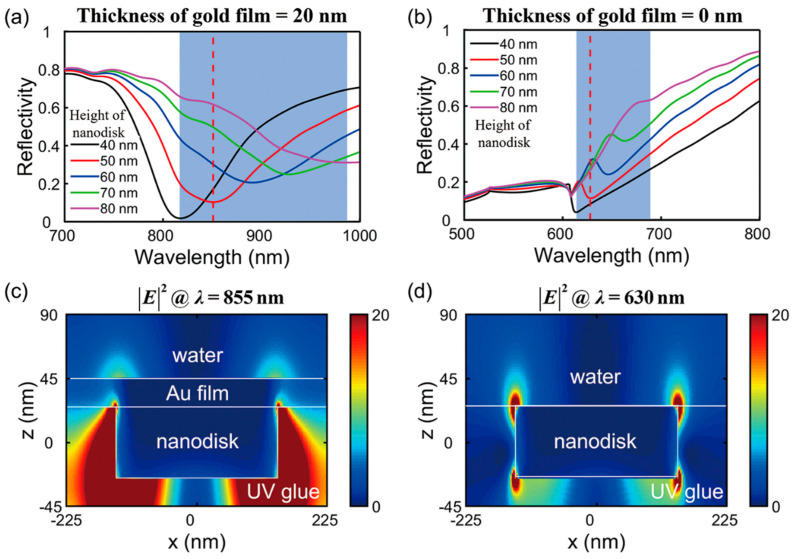
Simulated reflection spectra with various heights of Au nanodisk array for two comparative nanostructures: (**a**) Au nanodisk array/thin film coupled structure and (**b**) only the Au nanodisk array. The spatial distributions of the electric field in the *x–z* plane for plasmonic resonant dips of (**c**) Au nanodisk array/thin-film coupled structure and (**d**) Au nanodisk array. The wavelength positions of resonant dip corresponding to electric filed distributions in (**c**,**d**) are marked by vertical red dashed lines in (**a**,**b**).

In order to demonstrate the superiority of the designed plasmonic nanostructure, reflection spectra of a 280 nm diameter nanodisk array with and without a 20 nm thick Au thin film are analyzed comparatively. Figure 3a,b show simulated reflection spectra of Au nanodisk array/thin film coupled structure and only Au nanodisk array for various different heights of nanodisks, respectively, where the nanodisk arrays are embedded into UV curing glue with only the top surface directly touching the Au thin film or water solution. Obviously, the designed nanostructure possesses one resonant dip and its high depth always exceeds 0.6 in the studied wavelength range. As the height of nanodisks increases, the resonant dip has a large redshift, showing its remarkable capacity to adjust the resonance position. Notably, the redshift of the resonance position is accompanied by an increase in its linewidth and a decrease in its depth. Accordingly, the Au nanodisk array/thin film coupled structure with a relatively high height is beneficial to the design of a plasmonic nanostructure sensor. As a direct comparison, the depth of the resonant dip of the nanodisk array is shallow and no more than 0.2 regardless of the nanodisk’s height. Furthermore, in this case, the wavelength adjustment range of its resonant dip is also smaller compared to the nanodisk array/thin film coupled nanostructure.

To elucidate the generation mechanism of the plasmonic mode supported by the designed nanostructure, spatial profiles of electric field intensity in the *x–z* plane at resonant dips for two structures with a fixed height of nanodisk of 50 nm are depicted in Figure 3c,d. In the individual Au nanodisk array structure, diffractive coupling of localized surface plasmons at the interface between the top surface of the Au nanodisk and water solution is excited, usually called the Bloch-type surface plasmon polariton of the nanodisk array [33]. In addition, a Bloch-type surface plasmon polariton at the bottom surface of the Au nanodisk array also exists simultaneously due to light incidence from the bottom surface of the array. As shown in Figure 3c, the electric field profile in the designed structure at the wavelength of 855 nm is different from the case in only the Au nanodisk array. Due to the existence of thin Au film, the Bloch-type surface plasmon polariton at the bottom surface of the Au nanodisk array is dramatically enhanced. Concurrently, the localized near-field tunnels through the thin Au film to excite the plasmon mode at the top surface of the thin Au film. The thinner the top Au film, the stronger the plasmon mode at its top surface. 

### 3.2. The Evaluation of Bulk RI Sensitivity 

**Figure 4:**
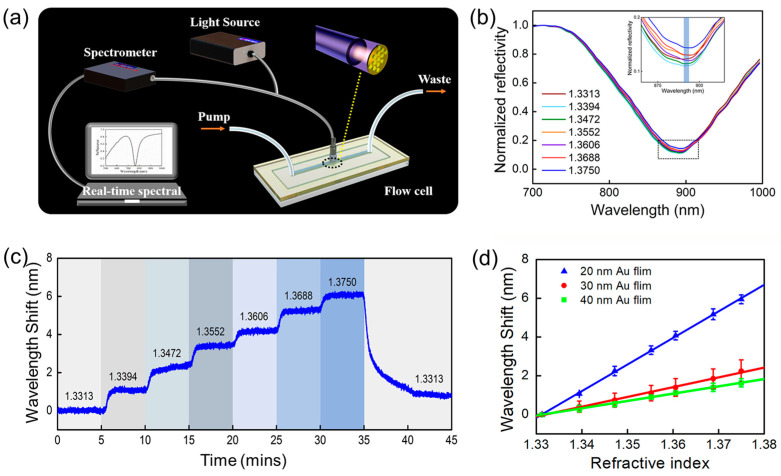
The quantitate evaluation of bulk RI sensitivity of the proposed nanodisk array/thin film-based fiber probe. (**a**) Schematic of experimental measurements setup for evaluating sensing performance of the proposed plasmonic fiber probe. (**b**) Normalized reflection spectra of the proposed Au nanodisk array/20 nm thick film-based fiber probe in the NaCl solution with RIs ranging from 1.3313 to 1.3750. (**c**) Wavelength redshift amount of resonant dip for different RIs of NaCl solution as a function of time. (**d**) Relationship between wavelength redshift amount of resonance dip and ambient RIs for Au thin film’s thickness of 20 nm, 30 nm, and 40 nm. Uncertainties in (**d**) are based on the results of three repeated measurements for every experimental data point.

The experimental measurement setup for sensing performance testing of the proposed plasmonic fiber probe is depicted in Figure 4a. A terminated reflection-type measurement system is constructed by a broadband halogen light source (HL-2000, Ocean Optics Inc., Orlando, FL, USA), a spectrometer (AvaSpec-Mini-NIR, Avantes Inc., Lafayette, CO, USA), and a fiber probe connected to a bifurcated optical fiber jumper. The fiber probe is connected to the common end of the fiber jumper and is integrated with a homemade flow cell. Analyte solutions are automatically injected into the flow cell using a peristaltic pump device with a constant flow rate of 1 mL/min at room temperature. The reflection spectra and wavelength positions of resonance dip are collected in real time and processed by a home-written LabVIEW program. Figure 4b shows the reflection spectra of the proposed Au nanodisk/20 nm thick film-based fiber probe immersed in different concentrations of NaCl solutions with corresponding RIs ranging from 1.3313 to 1.3750. The RIs of NaCl solutions are calibrated using an Abbe refractometer. Obviously, all reflection spectra under different RIs of NaCl solution show relatively good spectral features and have deep resonance dips. The wavelength of the resonant dip of this fiber probe has a stepwise redshift with the increase in the RIs of NaCl solutions. Figure 4c shows the real-time response wavelength shift of resonance dip for different RIs of NaCl solution. Obviously, when different RIs of NaCl solutions are switched, the response time of the sensor is a few tens of seconds, determined by the used flow cell. The smaller the liquid volume of the flow cell, the quicker the response time. The results of Figure 4c demonstrate that the fabricated plasmonic sensing probe enables quick responses to changes in the RI of the ambient surroundings while having good stability and response-recovery ability. What is more, the effect of the thickness of the top Au film on the bulk RI sensitivity of the plasmonic sensing probe is also investigated. Figure 4d summarizes the relationship between wavelength redshift amount of resonance dip and ambient RIs when the thickness of the top Au film is 20 nm, 30 nm, and 40 nm, respectively. By linearly fitting the data in Figure 4d, the bulk sensitivity of the plasmonic sensing probe is 137.28 nm/RIU, 50.47 nm/RIU, and 37.66 nm/RIU for Au film thicknesses of 20 nm, 30 nm, and 40 nm, respectively. As a result, the bulk RI sensitivity of the plasmonic sensing probe is negatively correlated with the thickness of the Au film, in conformity with the theoretical explanation in Figure 3. Therefore, taking into account the experimental conditions and preparation accuracy in our lab, a 20 nm thick Au film is selected for the fabrication of a nanodisk array/thin-film-based fiber probe.

### 3.3. Surface Sensitivity and Biomolecular Detection

**Figure 5:**
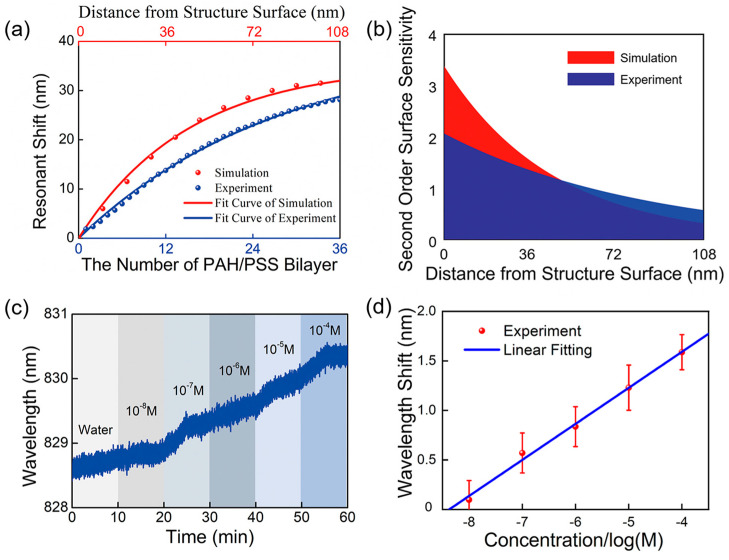
The quantitate evaluation of surface sensitivity of the proposed plasmonic fiber probe and its capacity to detect BSA biomolecules. (**a**) Simulated (red solid dot) and measured (blue solid dot) wavelength shift of resonant dip of the nanodisk/20 nm thick film-based fiber probe induced by increasing PAH/PSS bilayers. (**b**) Simulated (red zone) and measured (blue zone) surface sensitivity of resonance dip as a function of the distance from the structure surface. (**c**) Real-time monitoring of resonant wavelength for different concentrations of BSA aqueous solutions in ranging of 10^−8^ to 10^−4^ M. (**d**) The dependency of wavelength shift of resonant dip on the concentration of BSA biomolecules, where the blue solid line represents the linear fitting curve of wavelength shift. Each measurement for a given concentration of BSA biomolecule is repeated three times.

In order to comprehensively evaluate the sensing performance of the fabricated nanodisk/20 nm thick film-based fiber probe, the surface sensitivity of its resonant dip is first investigated numerically and experimentally by progressively increasing the number of self-assembly polyelectrolyte bilayers, where each bilayer consists of positively charged PAH and negatively charged PSS. In the simulation, each self-assembly polyelectrolyte bilayer is equivalent to a 2.9 nm thick dielectric layer with a high RI of 1.56. The simulated and measured results are summarized in Figure 5a. Notably, the wavelength of the resonance dip has a successive redshift and gradually approaches a plateau with an increasing number of polyelectrolyte bilayers. This is because the electric field intensity of the resonance dip decays exponentially with the increase in the distance from the top surface of the fiber tip, verifying the local enhancement feature of the excited plasmon mode in the proposed fiber probe. Furthermore, the simulated result is almost consistent with the measured one, except that the wavelength shift amount is somewhat larger than that in experiments with the increase in the number of PAH/PSS bilayers.

In order to further analyze the surface sensitivity of the resonant dip, its decay length is estimated by fitting the wavelength shift of the resonant dip in Figure 5a to the following equation:

(1) $$\Delta \lambda =\Delta \lambda_{\infty }\left(1-e^{-d/l_{d}}\right)$$

where Δ*λ* denotes the wavelength shift of the resonant dip, Δ*λ*_∞_ refers to the wavelength shift induced by an infinite thick dielectric layer, *l*_d_ represents the decay length of the resonant dip, and *d* is the distance from the structure surface. Second-order surface sensitivity, defined as the wavelength response to the change in the per unit thickness of the biomolecular layer and per unit RI, can be calculated by the following equation:

(2) $$S_{surf}=\frac{\partial^{2}\Delta \lambda }{\partial d\partial \Delta n}=\frac{\partial \Delta \lambda_{\infty }}{\partial \Delta n}\frac{\partial (1-e^{-d/l_{d}})}{\partial d}=\frac{\partial \Delta \lambda_{\infty }}{\partial \Delta nl_{d}}e^{-d/l_{d}}$$

where Δ*n* is the RI difference between the biomolecular layer and ambient surroundings, i.e., Δ*n* = 1.56 − 1.33 = 0.23. The theoretically simulated and experimentally measured surface sensitivity of the plasmonic sensing probe is depicted in Figure 5b on the basis of the fitted values of Δ*λ*_∞_ and *l*_d_ by Equation (1), respectively. Obviously, the second-order surface sensitivity of the excited plasmon mode decreases gradually with the increase in the distance from the structure surface. The closer the distance from the structure surface, the higher the second-order surface sensitivity, which effectively demonstrates local field characteristics of the resonance dip in the proposed plasmonic fiber probe. 

Except for the bulk RI sensitivity and surface sensitivity, the ability of the proposed plasmonic fiber probe to detect biomolecules as a practical biosensor is also investigated. In this experiment, BSA aqueous solutions with different concentrations ranging from 10^−8^ to 10^−4^ M are successively injected into the flow cell through the sensing surface, and the wavelength response of the resonant dip is collected and recorded in real time. As shown in Figure 5c, the wavelength of the resonant dip has a real-time redshift with the increase in the concentration of BSA molecules in the solution. Figure 5d summarizes the redshift amount of resonant dip as a logarithmic function of BSA molecules concentration. It is clear that the amount of wavelength shift of the resonance dip is linearly related to the logarithmic concentration of BSA biomolecules in the range of 10^−8^ to 10^−4^ M. Moreover, the limit of detection of the proposed plasmonic fiber probe can be calculated as 19.35 μM for BSA biomolecules, which demonstrates that the proposed fiber probe may have the potential for the detection of biomolecules. 

In order to clearly demonstrate the innovation and fabrication advantages of the proposed fiber tip sensor, Table 1 conducts a detailed comparison of our proposed fiber tip sensing probe and previously reported FOSPR sensor, including resonant structure, sensing performance, manufacturing technology, cost, and advantages. Obviously, the sensing performance of the proposed nanodisk array/thin film coupled resonance sensing probe is comparable to the case of previously reported FOSPR sensing tips [35,36]. Meanwhile, the proposed transferring technology possesses the advantages of high-efficiency, low-cost, and high-quality batch manufacturing as compared to other preparation methods.

## 4. Conclusions

In summary, we have experimentally demonstrated a Au nanodisk/thin film-based fiber probe, where the plasmon mode on the top Au film is excited by strong coupling between the Bloch-type surface plasmon polariton at the bottom surface of the Au nanodisk array and the top Au thin film. The planar Au film surface in the proposed plasmonic fiber probe is convenient for molecular modification in sensing detection as compared to an uneven surface. As a result, the excited resonance dip in this fiber probe demonstrates a bulk RI sensitivity of 137.28 nm/RIU and excellent surface sensitivity. In addition, we also successfully conduct real-time detection of BSA biomolecules at the detection limit of 19.35 μM, which demonstrates that the proposed plasmonic sensing probe may be applied to biochemical detection. Compared with traditional direct fabrication technology, such as FIB and EBL and other technologies, the UV curing glue transfer technology adopted here also has the advantages of high-efficiency, low-cost, and high-quality batch manufacturing. Due to its advantages of miniaturization and integration, the proposed plasmonic sensing probe is expected to further expand the practical application of FOSPR sensors and has a wide range of application prospects in biochemical detection, environmental monitoring, drug screening, and other fields.

## Figures and Tables

**Table 1:** Comparison of FOSPR sensing tips prepared by different processing techniques.

Reference	Resonant Structure	Sensing huichePerformance	Manufacturing Technology	Cost	Advantages
[26]	Au nanohole array with phase gradient	S ≈ 20 × 10^−12^ m/(ng mL^−1^) LOD ≈ 50 × 10^−12^ M	Focused ion beam (FIB)	High	High sensitivity
[35]	Au nanodot arrays	S = 196 nm/RIU LOD ≈ 6 pM	Electron beam lithography (EBL)	High	High LOD
[36]	Au nanodisk array	S = 187.4 nm/RIU	Ice-assisted electron beam lithography (iEBL)	High	Streamlined and ecofriendly
This work	Au nanodisk array/thin film	S = 137.28 nm/RIU LOD ≈ 19.35 μM	UV curing glue transfer technology	Low	Batch fabrication and convenience for molecular modification

## Data Availability

Data are available on request.
